# Nesfatin-1 suppresses interleukin-1β-induced inflammation, apoptosis, and cartilage matrix destruction in chondrocytes and ameliorates osteoarthritis in rats

**DOI:** 10.18632/aging.102711

**Published:** 2020-01-30

**Authors:** Lifeng Jiang, Kai Xu, Jin Li, Xindie Zhou, Langhai Xu, Zhipeng Wu, Chiyuan Ma, Jisheng Ran, Pengfei Hu, Jiapeng Bao, Lidong Wu, Yan Xiong

**Affiliations:** 1 Department of Orthopedics Surgery, The Second Affiliated Hospital, Zhejiang University School of Medicine, Hangzhou 310000, China; 2 Department of Orthopedics, The Affiliated Changzhou People’s Hospital of Nanjing Medical University, Changzhou 213003, China; 3 Department of Orthopedics Surgery, The Second Affiliated Hospital of Jiaxing University, Jiaxing 31400, China

**Keywords:** nesfatin-1, osteoarthritis, inflammation, apoptosis, matrix metalloproteinases

## Abstract

Osteoarthritis (OA) is a chronic degenerative joint disease, related to the overexpression of matrix metalloproteinases (MMPs), a disintegrin and metalloproteinase with thrombospondin motifs (ADAMTS), inflammation, and chondrocyte apoptosis. Nesfatin-1 is an adipokine, which plays an important role in the development of OA, especially in obese people. In the present study, cartilage degradation and apoptosis observed in OA patients was evaluated. Furthermore, the anti-inflammatory and anti-apoptotic effects of nesfatin-1, and its underlying in vitro and in vivo mechanisms were investigated. The results showed that nesfatin-1 increased significantly the expression of collagen type II alpha 1 chain (Col2a1), and reduced the expression of MMPs, ADAMTS5, cyclooxygenase (COX)-2, caspase-3, nitric oxide (NO), inducible nitric oxide synthase (iNOS), prostaglandin E2 (PGE2), interleukin (IL)-6, and chondrocyte apoptosis rate, which may be induced by IL-1β in rat chondrocytes. Furthermore, nesfatin-1 treatment prevented cartilage degeneration in the rat OA model. It was found that nesfatin-1 suppressed the IL-1β-induced activation of NF-κB, the mitogen-activated protein kinase (MAPK), and the Bax/Bcl-2 signal pathway in chondrocytes. These results suggest that in vivo nesfatin-1 could play a protective role in the development of OA and can be potentially used for its treatment.

## INTRODUCTION

Osteoarthritis (OA) is a common degenerative disease characterized by breakdown of the cartilage matrix, chondrocyte hypertrophy, inflammation of the synovial membrane, and osteophyte formation in the joints [1]. Old age, stress overload, oxidative stress, and genetic predisposition increases the risk of OA [2, 3]. To date, the treatment for OA has been primarily focused on the alleviation of pain and inflammation using nonsteroidal anti-inflammatory drugs (NSAIDs) or other agents. Therefore, there is a pressing need to find alternative treatment options for OA patients [4].

Chondrocytes are a single cellular component of the cartilage and play a key role in its degeneration. In OA, the degradation of the cartilage matrix and the apoptosis of chondrocytes are two crucial pathogenic events [5]. Matrix metalloproteinases (MMPs) and a disintegrin and metalloproteinase with thrombospondin motifs (ADAMTS) are the established primary mediators of cartilage degradation in OA; whereas, apoptosis has been observed in OA cartilage, suggesting its role in the pathogenesis of the disease [6]. In addition, this apoptotic process has been found to be correlated with cartilage damage and hypocellularity [7]. Therefore, apoptosis has become a potential target for the treatment of OA and its understanding is essential for the design of new therapeutics [8, 9].

Interleukin (IL)-1β is a crucial inflammatory factor that plays a pivotal role in the pathogenesis of OA [10, 11]. It is known to trigger chondrocyte apoptosis and has thus been widely used as a chondrocyte apoptosis-inducing agent [12]. Therefore, chondrocytes treated with IL-1β provide a useful model of OA chondrocytes. Previous studies have shown that IL-1β can trigger a cascade of inflammatory events [3], induce the expression of MMPs, and lead to cartilage matrix degradation in OA [4]. Additionally, IL-1β can induce the production of nitric oxide (NO) and prostaglandin E2 (PGE2), two inflammatory mediators which have also been associated with cartilage degradation in OA.

Nesfatin-1, an 82-amino-acid peptide, is found in abundance in adipose, nervous, and digestive tissues [13]. Previous studies have shown that nesfatin-1 has multifunctional effects, such as being anorexigenic [14], stress and blood pressure modulation [15, 16], anti-inflammatory action [17], protection from ischemia/reperfusion injury, and control of the reproductive axis [18, 19]. It has been reported that adiponectin, which is another adipokine, could protect rat chondrocytes from hydrogen peroxide-induced apoptosis by inducing autophagy [20]. On the other hand, leptin has been found to promote chondrocyte apoptosis in the pathogenesis of OA [21]. These studies indicated that adipokines have an effect on chondrocyte apoptosis and are involved in its protection. Recent studies have shown that nesfatin-1 acts as a protective agent, due to its anti-apoptotic actions on the myocardium and neurons [22–24]. In contrast, nesfatin-1 was found to elevate MMP-2/MMP-9 levels in proliferation and migration of vascular smooth muscle cells (VSMCs), resulting in neointimal hyperplasia [25]. A correlation between nesfatin-1 and MMP-2 has been observed in rheumatoid arthritis [26]. Furthermore, nesfatin-1 can reduce the expression of nitric oxide (NO) and prostaglandin E2 (PGE2) by suppressing inducible nitric oxide synthase (iNOS) and cyclooxygenase (COX)-2, thereby protecting the gastric mucosa [27]. These results have suggested that nesfatin-1 is not only related to MMPs and anti-inflammatory players, but also has anti-apoptotic effects. Our previous study demonstrated that the level of nesfatin-1 in the articular cartilage of OA patients increases [28]. However, the effect of nesfatin-1 on chondrocyte apoptosis, inflammation, and matrix destruction in OA and its molecular mechanism of action remain unclear.

Due to their anti-apoptotic and anti-inflammation properties, adipokines have become attractive interventions in attenuating IL-1β-induced chondrocyte apoptosis. In view of this, the present study aimed to investigate the potential effect of nesfatin-1 on IL-1β-stimulated chondrocytes, and to elucidate its underlying mechanism.

## RESULTS

### Cartilage degradation, apoptosis, and inflammation was observed in OA

**Figure 1:**
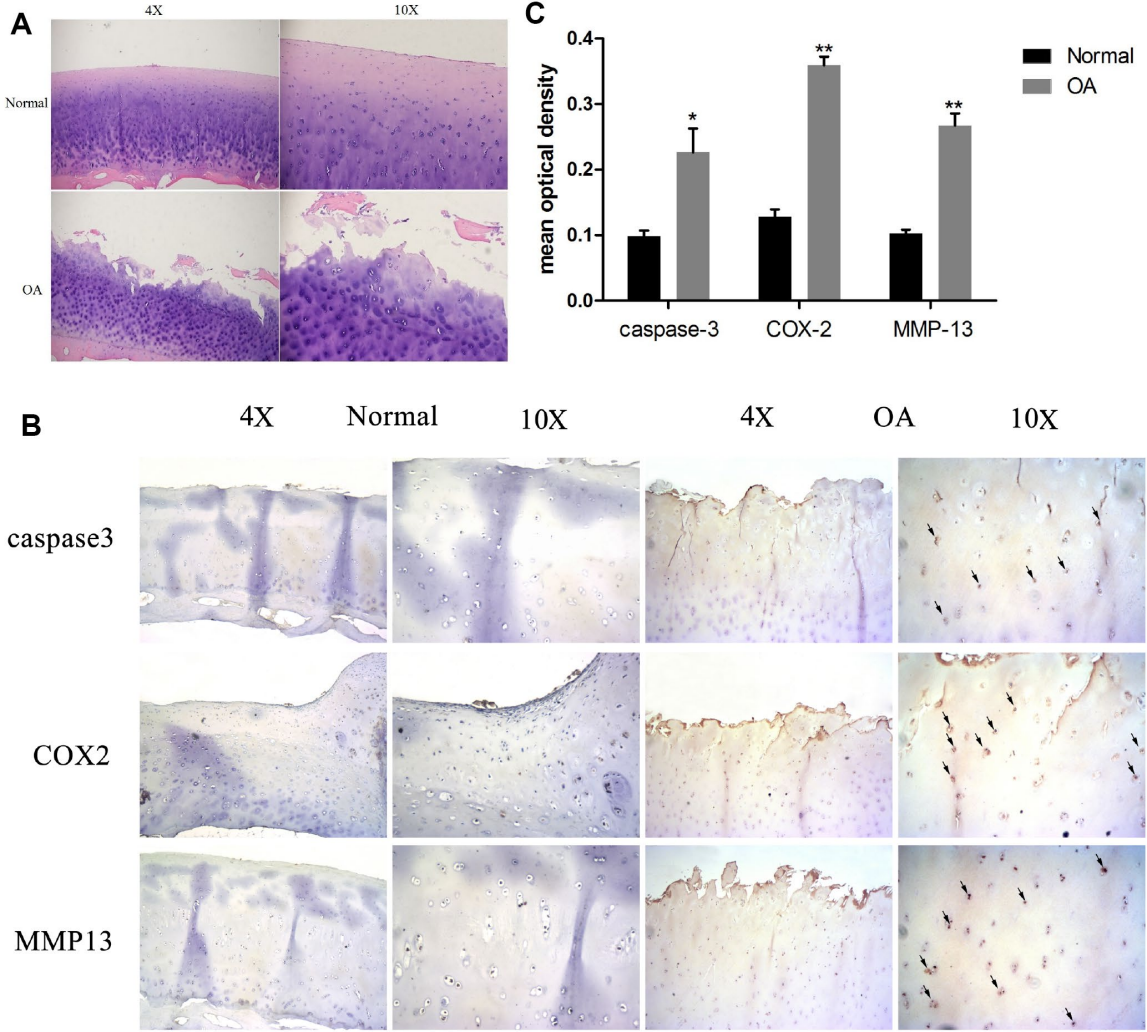
**Chondrocyte apoptosis, inflammation, and matrix degradation in the OA samples.** (**A**) Representative images of H&E staining; (**B**) Representative images of immunohistochemistry; (C60) Quantitative optical density analysis of immunohistochemistry. Data represent the mean ± SD (n=5) and were analyzed by one-way analysis of variance followed by Tukey's post hoc test. ** *p* < 0.01 and * *p* < 0.05 versus normal samples.

Normal and OA articular cartilage samples were used for histological and immunohistochemical analysis. As Figure 1A shows, the H&E staining revealed that cartilages in the OA samples were degraded severely (surface wear, matrix fissuring, and decreased number of chondrocytes) compared to normal tissues. As per the IHC results (Figure 1B and 1C), the signal of MMP-13, a well-known cartilage degradation-related protein, was remarkably increased. Similarly, the apoptosis-related protein caspase-3, and the inflammatory-related marker COX-2 were also significantly increased. These results indicated that cartilage degradation, apoptosis, and inflammation were evident in the OA samples as compared to the normal samples.

### Effect of various nesfatin-1 concentrations on IL-1β-treated chondrocytes

**Figure 2:**
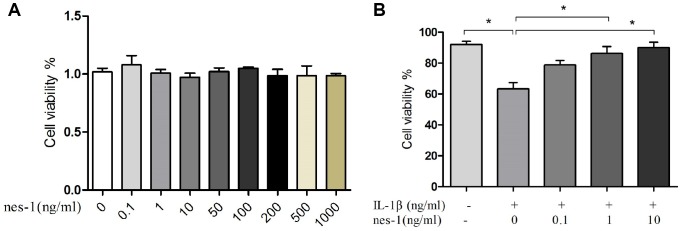
**Effect of nesfatin-1 and IL-1β on cell viability.** (**A**) Cells treated with increasing concentrations of nesfatin-1 (0, 0.1, 1 to 1000 ng/mL) for 24 h. (**B**) Pre-treatment with nesfatin-1 for 2 hours, followed by IL-1β treatment for 24 h. Chondrocyte viability was evaluated via CCK-8 analysis. Data represent the mean ± SD (n=3) and were analyzed by one-way analysis of variance followed by Tukey's post hoc test.* *p* < 0.05 versus IL-1β-treated alone samples.

The effect of nesfatin-1 on cell survival is shown in Figure 2. After co-culturing for 24 h, the cytotoxicity of nesfatin-1 was measured using CCK-8 assay. As it can be seen in Figure 2, nesfatin-1 exerted no significant cytotoxic effect at concentrations ranging from 0.1–1000 ng/ml. Compared to cells in the control group (0.2% DMSO), the cells treated with IL-1β (10 ng/mL) showed significantly reduced cell viability. On the other hand, compared to the cells treated with IL-1β, the cells treated with nesfatin-1 showed better viability. These results suggested that the IL-1β treatment reduced significantly the cell viability of chondrocytes, while nesfatin-1 exerted a protective effect on OA chondrocytes.

### Nesfatin-1 suppresses IL-1β-induced apoptosis in chondrocytes

**Figure 3:**
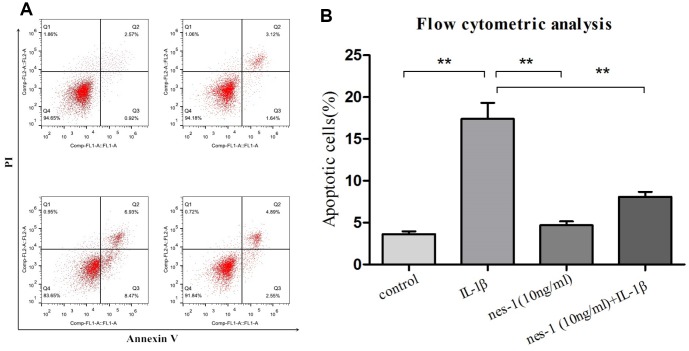
**Apoptosis rate determination using flow cytometry of nesfatin-1- and IL-1β-treated cells.** (**A**) Cells pre-treated with nesfatin-1 (10 ng/mL) for 2 hours, followed by IL-1β treatment for 24 hours, and analyzed by flow cytometry. (**B**) Flow cytometric analysis. IL-1β-stimulation alone significantly increased the percentage of apoptotic chondrocytes. Annexin V- and PI-positive cells were markedly decreased among nesfatin-1 pre-treated cells. Data represent the mean ± SD (n=3) and were analyzed by one-way analysis of variance followed by Tukey's post hoc test. ** *p* < 0.01 versus the IL-1β group.

**Figure 4:**
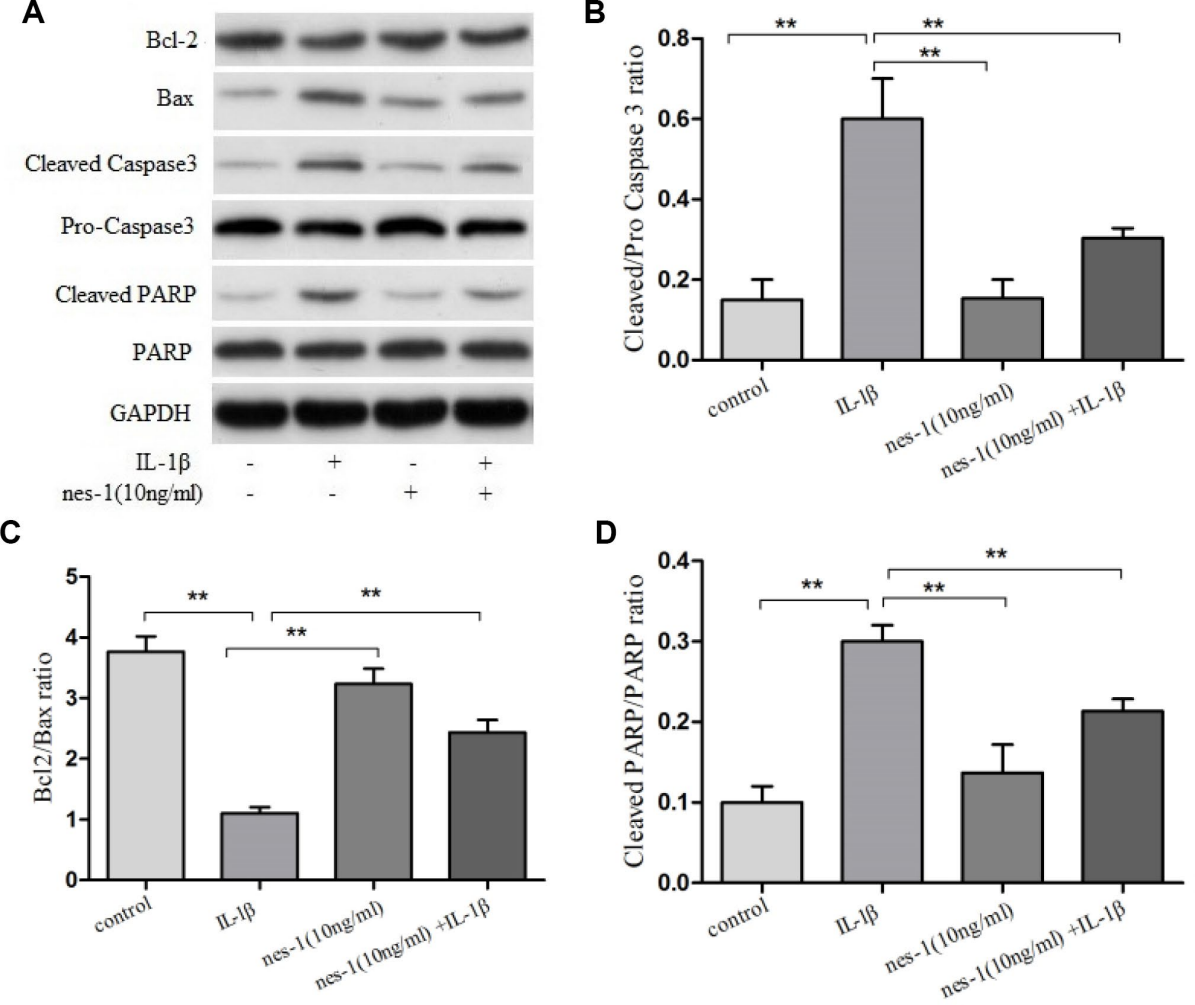
**Effect of nesfatin-1 on IL-1β-induced activation of apoptosis in rat chondrocytes.** (**A**) Chondrocytes were treated with nesfatin-1 on IL-1β-induced chondrocytes for 6 h and the protein levels of Bcl-2, Bax, cleaved caspase-3, pro-caspase-3, cleaved PARP, and PARP were evaluated via western blot analysis. (**B**–**D**) Quantitative analysis of western blot in rat chondrocytes. Data represent the mean ± SD (n=3) and were analyzed by one-way analysis of variance followed by Tukey's post hoc test. ** p < 0.01 versus the IL-1β group.

Although the mechanism of nesfatin-1 in protection of OA chondrocytes has not been elucidated yet, its anti-apoptotic effect in ovarian cells has been previously reported. Based on this, it was speculated that nesfatin-1 may also exert this effect on cartilages. Therefore, a dose of 10 ng/mL nesfatin-1 was selected for subsequent experiments and its effect on the apoptosis of IL-1β-stimulated chondrocytes was evaluated. As expected, the IL-1β-treated group showed a marked increase in the number of apoptotic chondrocytes; whereas, this effect was partially rescued with the nesfatin-1 treatment (Figure 3). The effect of nesfatin-1 on apoptotic markers was further evaluated using western blotting analysis. Caspase-3 and the downstream effector proteins are typically the major players involved in apoptosis. As it can be seen in Figure 4A, 4B and 4D, the nesfatin-1 treatment significantly reduced the levels of cleaved caspase-3 and cleaved PARP, compared to those in the IL-1β group. These results demonstrated that nesfatin-1 inhibited the IL-1β-induced apoptosis of chondrocytes.

### Nesfatin-1 decreased the MMPs and ADAMTS5, and increased the Col2a1 expression in chondrocytes

**Figure 5:**
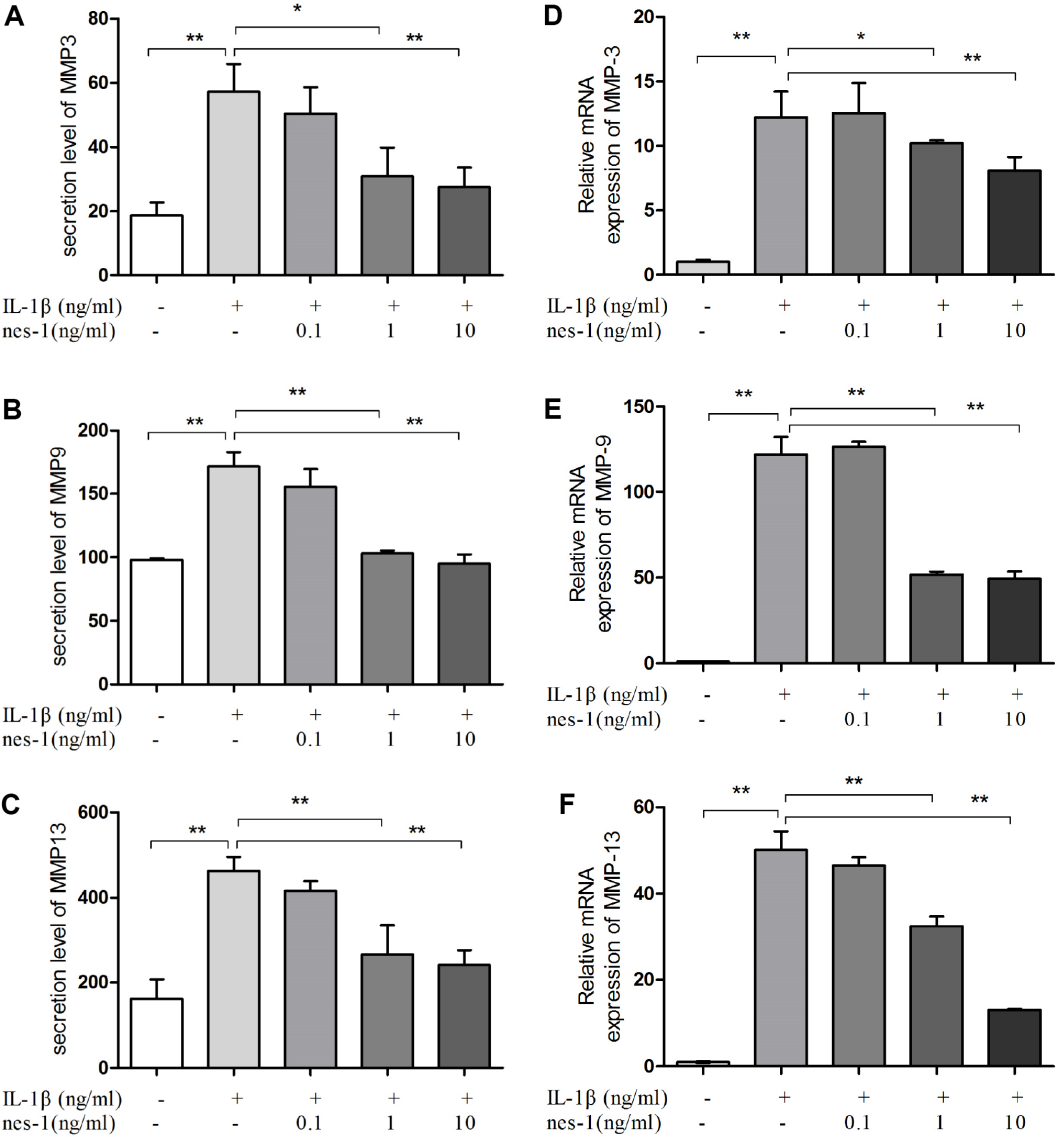
**Nesfatin-1 suppressed the IL-1β-induced MMP-3, MMP-9, and MMP-13 expression in rat chondrocytes and the secretion level in the supernatant.** Chondrocytes were treated with different concentrations of nesfatin-1 on IL-1β-induced chondrocytes for 24 h. The secretion level of MMPs was evaluated by ELISA (**A**, **B**, and **C**) and the mRNA level of MMPs was evaluated via real-time PCR (**D**, **E**, and **F**). The values are expressed as mean ±SD (n=3) and were analyzed by one-way analysis of variance followed by Tukey's post hoc test. **p < 0.01 versus the IL-1β group.

**Figure 6:**
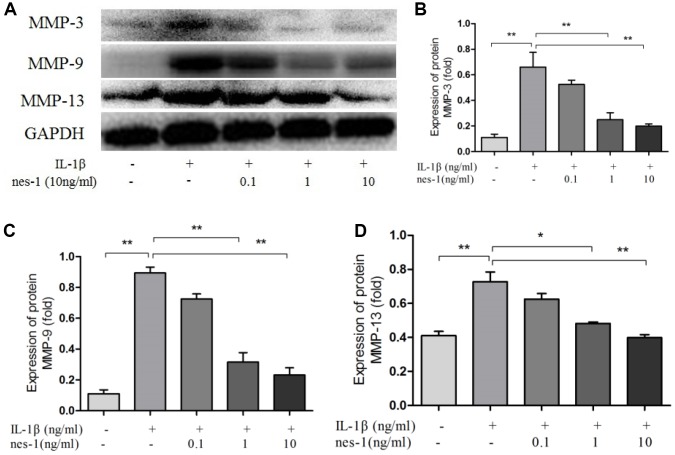
**Nesfatin-1 suppressed the IL-1β-induced MMP-3, MMP-9, and MMP-13 expression in rat chondrocytes.** Chondrocytes were treated with different concentrations of nesfatin-1 on IL-1β-induced chondrocytes for 24 h. The protein level of MMPs was evaluated via western blot analysis (**A**, **B**, **C**, and **D**). The values are expressed as mean ±SD (n=3) and were analyzed by one-way analysis of variance followed by Tukey's post hoc test. **p < 0.01 versus the IL-1β group.

**Figure 7:**
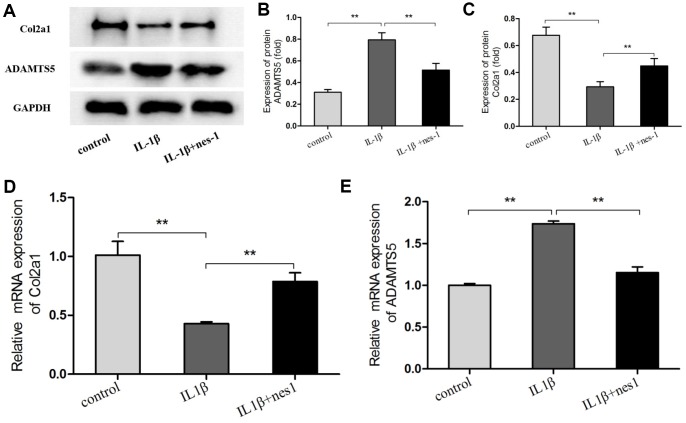
**Nesfatin-1 suppressed the IL-1β-induced ADAMTS5 expression and reversed IL-1β-induced Col2a1 degradation in rat chondrocytes.** Chondrocytes were treated with different concentrations of nesfatin-1 on IL-1β-induced chondrocytes for 24 h. The mRNA levels of ADAMTS5 and Col2a1 were evaluated via real-time PCR (**D**, **E**) and the protein levels of ADAMTS5 and Col2a1 were evaluated via western blot analysis (**A**, **B**, and **C**). The values are expressed as mean ±SD (n=3) and were analyzed by one-way analysis of variance followed by Tukey's post hoc test. **p < 0.01 versus the IL-1β group.

The cartilage matrix degradation induced by IL-1β is mediated by catabolic enzymes such as MMPs, including MMP-3, MMP-9, MMP-13, and ADAMTS5, which can cleave the main constituent of the extracellular matrix. Col2a1 is the major component of the cartilage matrix, the degradation and reduction of which are frequently observed in OA cartilage [29, 30]. Therefore, the effect of nesfatin-1 on the gene and protein expression of these enzymes was evaluated using real-time PCR and western blot analysis, and the secretion level in the supernatant was detected. Our results indicated that pre-treatment with nesfatin-1 can significantly inhibit secretion (Figure 5A–5C), mRNA level (Figure 5D–5F), and protein expression (Figure 6A–6D) of MMP-3, MMP-9, MMP-13, and ADAMTS5 (Figure 7A, 7B, 7E), and increase the mRNA level and protein expression of Col2a1 (Figure 7A, 7C, 7D), as compared to IL-1β treatment chondrocytes.

### Nesfatin-1 decreased the iNOS, COX-2, and IL-6 expression and the NO and PGE2 production in chondrocytes

**Figure 8:**
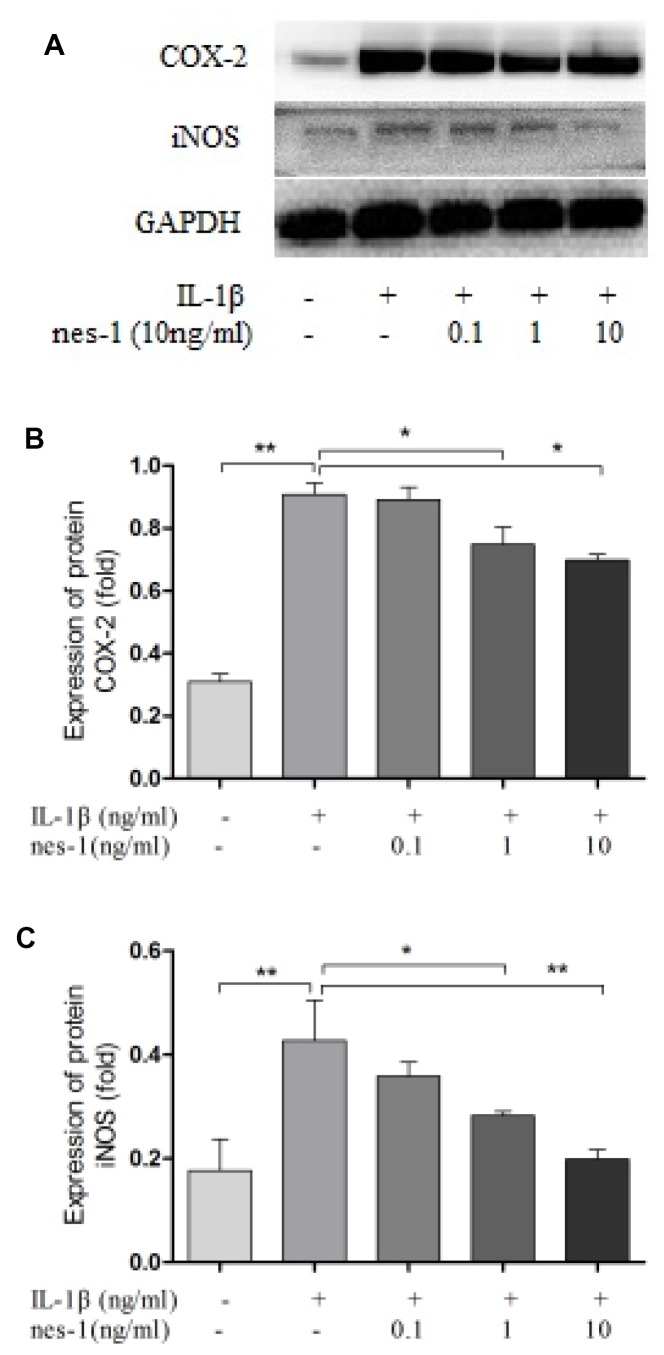
**Nesfatin-1 suppressed the IL-1β-induced iNOS and COX-2 expression in rat chondrocytes.** Chondrocytes were treated with different concentrations of nesfatin-1 on IL-1β-induced chondrocytes for 24 h. The protein levels of COX-2 and iNOS were evaluated via western blot analysis (**A**, **B**, and **C**). The values are expressed as mean ±SD (n=3) and were analyzed by one-way analysis of variance followed by Tukey's post hoc test. *p<0.05, **p < 0.01 versus the IL-1β group.

**Figure 9:**
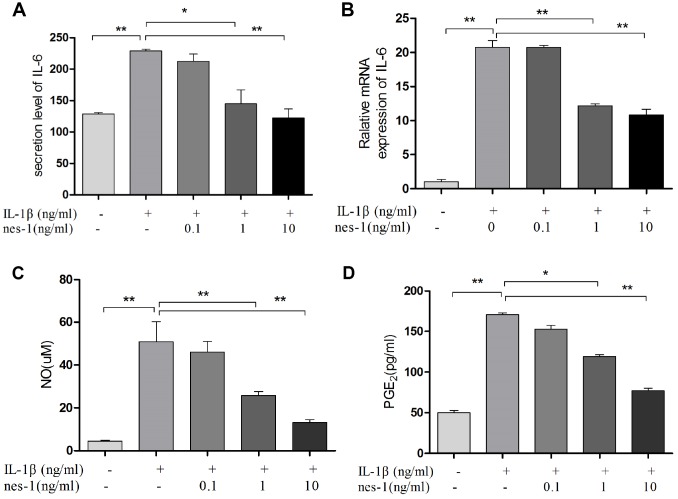
**Nesfatin-1 suppressed the IL-1β-induced IL-6, NO, and PGE2 expression in rat chondrocytes and the supernatant.** Chondrocytes were treated with different concentrations of nesfatin-1 on IL-1β-induced chondrocytes for 24 h. The secretion level of IL-6 in the supernatant was evaluated via ELISA (**A**) and the mRNA level of IL-6 was evaluated via real-time PCR (**B**). The NO level was evaluated via Griess reaction (**C**) and the PGE2 level was evaluated via ELISA (**D**). The values are expressed as mean ±SD (n=3) and were analyzed by one-way analysis of variance followed by Tukey's post hoc test. *p<0.05, **p < 0.01 versus the IL-1β group.

Nitric oxide, PGE2, and IL-6 are inflammatory mediators, which are involved in the degradation of articular cartilage. IL-1β can up-regulate the expression of iNOS and COX-2, as well as the production of NO and PGE2 in OA. The inhibition of NO or PGE2 can attenuate the progression of OA. Hence, the effect of nesfatin-1 on iNOS and COX-2 gene expression and the protein levels in IL-1β-induced chondrocytes were investigated, and the secretion level of IL-6 in the supernatant was detected. The results demonstrated that nesfatin-1 suppressed the protein levels of iNOS and COX-2 (Figure 8A–8C), as well as the inflammatory mediator IL-6 (Figure 9A, 9B). The production of PGE2 was determined by ELISA and the production of NO was assessed by Griess reaction. Nesfatin-1 treatment resulted in significant down- regulation of NO and PGE2 production induced by IL-1β in chondrocytes (Figure 9C, 9D).

### Nesfatin-1 elicited anti-apoptotic effect via the Bax/Bcl-2 pathway

The pro-apoptotic protein Bax and the anti-apoptotic protein Bcl-2 are two of the prominent apoptosis proteins. The balance of Bax and Bcl-2 plays an important role in cell apoptosis and survival. In order to explore the mechanism of nesfatin-1 in protecting chondrocytes from IL-1β-induced apoptosis, the levels of these proteins were evaluated (Figure 4A, 4C).

Western blot analysis revealed that the expression level of Bcl-2 declined, while that of Bax increased in the IL-1β-treated group, as compared to the nesfatin-1 pre-treated group (*p* < 0.01).

### MAPK signaling pathway is involved in nesfatin-1 inhibition of MMPs expression

**Figure 10:**
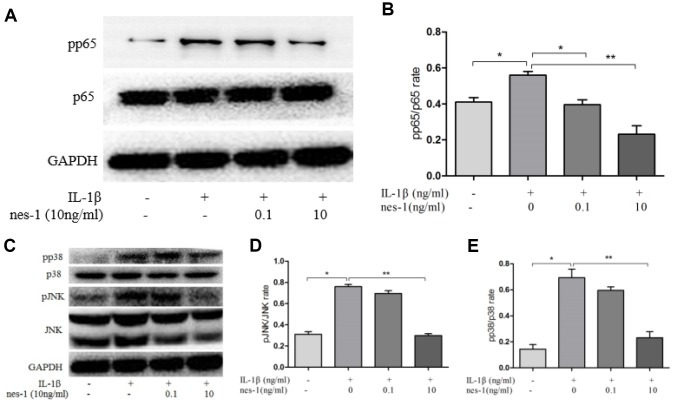
**Effect of nesfatin-1 on the IL-1β-induced NF-κB and MAPK activation in rat chondrocytes.** (**A**, **C**). Chondrocytes were treated with different concentrations of nesfatin-1 for 6 h after IL-1β-induction. The protein levels of p38, phosphor-p38, JNK, phosphor-JNK, p65, and phosphor-p65 were evaluated via western blot analysis (**B**, **D**, and **E**). Quantitative analysis of western blot in rat chondrocytes. The values are expressed as mean ±SD (n=3) and were analyzed by one-way analysis of variance followed by Tukey's post hoc test.*p < 0.05, **p < 0.01 versus the IL-1β group.

The MAPK signaling pathway plays an important role in the pathophysiology of OA, especially in the regulation of MMPs expression. Previous studies have shown that nesfatin-1 can affect the activation of MAPK. Thus, the effects of nesfatin-1 on MAPK in IL-1β-stimulated chondrocytes were investigated. Phosphorylation of p38 and JNK was increased by IL-1β stimulation, while it was inhibited by the treatment with nesfatin-1 (Figure 10C–10E).

### NF-κB signaling pathway is involved in nesfatin-1 inhibition of iNOS and COX-2 expression

The NF-κB signaling pathway is thought to be involved in the inflammatory progression of OA. Since p65 is an indicator of NF-κB activation after IL-1β stimulation, the protein levels of p65 in the nuclear and cytoplasmic fractions were checked. The results showed that pp65 was significantly increased after IL-1β stimulation, however, nesfatin-1 inhibited its phosphorylation (Figure 10A, 10B).

### Nesfatin-1 regulates cartilage degradation, apoptosis, and inflammation in rat OA models

**Figure 11:**
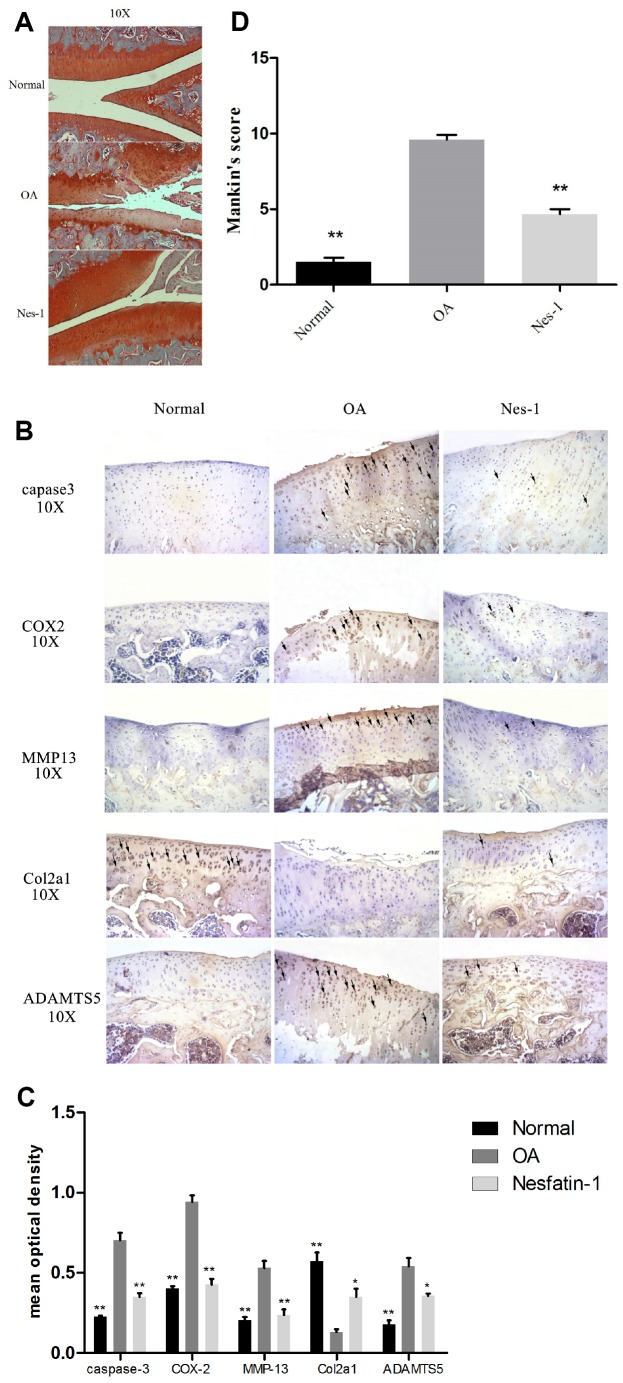
**Specimen evaluation in animal models.** (**A**, **D**) Representative images of safranin-O-stained rat knee joint sections with different treatments and the related Mankin scores of the three groups. Scale bar=200 μm. Data represent the mean ± SD (n = 30) and were analyzed by one-way analysis of variance followed by Tukey's post hoc test. ** *p* < 0.01 versus the OA group. (**B**) Representative immunohistochemistry images against MMP-13, COX-2, caspase-3, ADAMTS5, and Col2a1. Scale bar=200 μm; (**C**) Quantitative optical density analysis of immunohistochemistry for the different groups. Data represent the mean ± SD (n = 30) and were analyzed by one-way analysis of variance followed by Tukey's post hoc test. **p < 0.01 versus the OA group.

Given the findings on nesfatin-1, a rat OA model was developed to evaluate the corresponding effects *in vivo*. Nesfatin-1 was injected into the knee joints of OA rats and the cartilage was collected for histological evaluation. Reduced Safranin O staining and severe cartilage destruction was evident in the OA group compared to the normal group. Intra-articular injection of nesfatin-1 remarkably alleviated the surgicalresection-induced cartilage destruction (Figure 11A–11D). These results were additionally validated using the Mankin score, which suggested that the nesfatin-1treatment significantly attenuated OA in rats compared to the OA group (*p* < 0.01).

Immunohistochemical experiments for cartilage degradation-, apoptosis-, and inflammation-related proteins demonstrated that the expression of MMP-13, COX-2, caspase-3, and ADAMTS5 in the control group was significantly lower than that in the OA group (p < 0.01), while, that in the nesfatin-1 group was also remarkably lower than that in the OA group (p < 0.01; Fig. 11B). The relative optical density of these parameters in the OA group increased significantly compared to the nesfatin-1 group (p < 0.01, Fig. 11C), which was similar to that observed in the control group. On the contrary, the expression of Col2a1 in the OA group was significantly lower than that in the control group (p < 0.01), while, this was reversed by the nesfatin-1 treatment (p < 0.01; Fig. 11B). According to the above results, it can be concluded that nesfatin-1 ameliorates cartilage degradation, apoptosis, and inflammation in rat OA models.

## DISCUSSION

Osteoarthritis is the most common joint disease globally, and its prevalence increases in an age-associated manner [31]. However, its underlying mechanism remains unknown, and thus, its treatment is a challenge. So far, many studies have explored the association between adipokine, obesity, and OA [32–34]. For instance, leptin has been found to be significantly increased in OA patients [35] and it has catabolic and proinflammatory effects in chondrocytes [36]. Nevertheless, leptin has been shown to play a protective role for chondrocytes in OA [37]. Thus, the relationship between adipokines, OA, and its mechanism requires further investigation.

In our previous study, it was found that nesfatin-1 is significantly increased in OA patients [28]. To further explore the underlying mechanism, the present experimental study was conducted and some attractive results were found.

It is well established that cartilage degeneration plays a central role in OA development. The synthesis and degradation of the cartilage matrix in normal joints are in dynamic balance. MMPs and ADAMTS5 participate in cartilage degradation in OA. It has been found that and causes cartilage destruction by MMP upregulation [38]. Therefore, inhibition of the MMPs and ADAMTS5 expression and chondrocyte inflammation has been shown to have a therapeutic effect in OA. This understanding is consistent with our H&E and IHC staining results of human samples, where marked cartilage degeneration, matrix destruction, and a significant increase in MMP-13, COX-2, and caspase-3 in OA cartilage compared to that of normal cartilage was observed. In *in vitro* studies, we observed that nesfatin-1 treatment at different concentrations (0, 0.1,1…1000ng/mL) did not affect the viability of rat chondrocytes. It was observed that the IL-1β treatment significantly reduced the viability of chondrocytes, while the nesfatin-1 treatment reversed the cell survival, exerting its protective effect on chondrocytes in OA. This phenomenon was also evident by the flow cytometry results, where nesfatin-1 reduced the rate of IL-1β-induced apoptosis of chondrocytes. Next, our data revealed that nesfatin-1 significantly reduced the IL-1β-induced expression of inflammatory mediators, including iNOS, COX-2, NO, IL-6, and PGE2, and matrix-degrading enzymes, such as MMP-3, MMP-9, MMP-13, and ADAMTS5. Meanwhile, nesfatin-1 could reverse the IL-1β-induced degradation of Col2a1, suggesting that nesfatin-1 exerts a protective action via decreasing these players in the chondrocytes of IL-1β induced rats.

Moreover, *in vivo* immunohistochemistry indicated that, in the nesfatin-1-treated group, COX-2, MMP-13, Caspase 3, and ADAMTS5 were significantly decreased, while Col2al was increased compared to the OA group. Histological analysis indicated that nesfatin-1 could reverse the hypocellularity and structural damage observed in the OA model and ameliorate cartilage erosion. These results demonstrated that nesfatin-1 has anti-inflammatory and cartilage-protective effects in OA.

Two key signaling pathways, namely NF-κB and MAPK, are involved in OA development [39, 40]. In rest status, p65 resides with its inhibitor IKB in the cytoplasm. However, when activated by inflammatory mediators like IL-1β, p65 gets phosphorylated, translocates into the nucleus, and further up-regulates multiple inflammation-related genes, such as MMPs, COX-2, iNOS, IL-6, and PGE2 [41]. Our results revealed that nesfatin-1 inhibited p65 phosphorylation and nuclear translocation, indicating a suppressive role of the NF-κB pathway in chondrocytes. Furthermore, p38 and JNK are primarily involved in the MAPK pathway, which can be activated by phosphorylation. Therefore, in this study, the effects of nesfatin-1 on the IL-1β-induced phosphorylation of JNK and p38 were examined. Nesfatin-1 significantly suppressed the phosphorylation of p38 and JNK in IL-1β-induced rat chondrocytes, indicating a suppressive role of the MAPK pathway in chondrocytes.

Apoptosis typically occurs in osteoarthritic cartilage, while caspases and Bcl-2 family proteins are key molecules involved in this process [42]. A Bcl-2-like protein 4, Bax, found in the cytosol, is involved in the initiation of apoptosis. The Bcl-2/Bax ratio determines whether a cell will survive or undergo apoptosis. In the OA group, the Bcl-2/Bax ratio was found significantly decreased compared to that in the control group [43]. The decreased Bcl-2/ Bax ratio leads also to the activation of caspase-3 [44]. Our data demonstrated that nesfatin-1 can suppress the increase of the Bcl-2/Bax and cleaved caspase 3/pro-caspase 3 ratios caused by IL-1β stimulation. Similar results were observed for cleaved-PARP, which facilitates cellular disassembly and serves as a marker for cells undergoing apoptosis [45]. The expression of cleaved-PARP was increased with IL-1β stimulation and significantly decreased after pre-treatment with nesfatin-1. These results suggest that nesfatin-1 has an anti-apoptotic effect on OA chondrocytes.

Based on these *in vitro* results, the hypothesis that intra-articular nesfatin-1 injection in OA rats can maintain the extracellular matrix homeostasis and attenuate the progression of the disease was further validated *in vivo*. The subsequent experimental results suggested that the margin of the matrix was smooth and complete, and chondrocyte apoptosis, as well as inflammation, were alleviated in the nesfatin-1-treated group as compared to the OA group.

However, there are a few limitations to this study. Although this is a classic method for establishing an OA model, it differs from the clinical development process of OA, which may be caused by many factors. Secondly, this study focused on the effects of nesfatin-1 on chondrocytes and cartilage, and excluded its effects on other cells, like synoviocytes, or tissues, like the subchondral bone and meniscus. Therefore, the impact of nesfatin-1 on OA needs to be further studied in-depth.

In conclusion, our results demonstrated that nesfatin-1 can inhibits MMP expression and chondrocyte inflammation through the suppression of both the NF-κB and MAPK signal pathways. Furthermore, it can reduce the apoptosis in rat chondrocytes via the Bax/Bcl-2 pathway. Animal experiments revealed the protective effect of nesfatin-1 in OA cartilage. This study provides a novel understanding of the mechanism of adipokine from the OA perspective, and explores its potential therapeutic application.

## MATERIALS AND METHODS

### Reagents

Recombinant human nesfatin-1 and recombinant rat IL-1β were purchased from Sigma–Aldrich (St Louis, MO, USA). IL-1β (1 mg/mL) was dissolved in double-distilled water and nesfatin-1 powder (1 mg/mL) was dissolved in phosphate buffer saline (PBS). Growth medium Dulbecco's modified Eagle medium (DMEM)/F12 and fetal bovine serum (FBS) were also procured from Sigma–Aldrich (St Louis, MO, USA).

The following monoclonal antibodies were obtained: anti-Bcl-2 (rabbit monoclonal antibody (mAb); cat. no. ab194583; Abcam), anti-Bax (rabbit mAb; cat. no. ab32503; Abcam), anti-procaspase-3 (rabbit mAb; cat. no. ab90437; Abcam), anti-cleaved caspase-3 (rabbit mAb; cat. no. 9661; Cell Signaling Technology, Inc), anti-PARP (rabbit mAb; cat. no. ab194586; Abcam), anti-cleaved-PARP (rabbit mAb; cat. no. ab32064; Abcam), anti-Glyceraldehyde 3-phosphate dehydrogenase (GAPDH) (rabbit mAb; cat. no. ab181602; Abcam), anti-MMP-3 (rabbit mAb; cat. no. ab52915; Abcam), anti-MMP-9 (rabbit mAb; cat. no. ab76003; Abcam), anti-MMP-13 (rabbit mAb; cat. no. sc-30073; Santa Cruz Biotechnology, Inc.), anti-Col2a1 (rabbit mAb; cat. no. ab34712; Abcam), anti-ADAMTS5 (rabbit mAb; cat. no. ab41037), anti-iNOS (rabbit mAb; cat. no. ab3523; Abcam), anti-COX-2 (D5H5; rabbit mAb; cat. no. 12282; Cell Signaling Technology, Inc), anti-pp65 (Ser536; rabbit Ab; cat.no. 3031; Cell Signaling Technology, Inc.), anti-p65 (p65; C22B4; rabbit mAb; cat. no. 4764S; Cell Signaling Technology, Inc), anti-pp38 (rabbit mAb;cat. no. 4511; Cell Signaling Technology, Inc), anti-p38 (rabbit mAb;cat. no. 9212; Cell Signaling Technology, Inc), anti-pJNK (rabbit mAb; cat. no. 4668; Cell Signaling Technology, Inc), anti-JNK (rabbit mAb; cat. no. 9258; Cell Signaling Technology, Inc). A cell-counting kit-8 assay (CCK-8) was obtained from Dojindo Laboratories (Kumamoto, Japan). Hematoxylin and eosin stain (H&E) was obtained from Beyotime Biotechnology (Shanghai, China). ELISA kits were obtained from Shanghai NuoYuan industrial co. LTD: MMP3 (NY54968), MMP9 (NY25976), MMP13 (65967), IL-6 (NY72941).

### Collection of human articular cartilage samples, and H&E and immunohistochemical staining

This study was approved by the local ethics committee and a written informed consent was obtained from each volunteer. Five OA cartilage specimens were collected from patients (aged 63.5± 4.5 years) who underwent total knee joint replacement surgery at the Second Affiliated Hospital, Zhejiang University School of Medicine, China. The distinctively worn area and surrounding cartilage were selected. In general, 4 specimens (lateral and medial femoral condyle and lateral and medial tibial plateau) with surface abrasion can be obtained from one OA patient sample. Three normal cartilage specimens were collected from patients with traumatic amputation without OA. Each sample was fixed in 4% paraformaldehyde, decalcified with 10% formic acid for three months, buffered at pH 7.4, dehydrated through a series of ethanol solutions, embedded in paraffin, cut into 8-10 3-*μ*m-thick slices, and subsequently stained. For H&E staining, the samples were first stained with hematoxylin for 5 min, washed with water, stained with eosin for 5 min, and then dehydrated with xylene and mounted in neutral balsam. For immunohistochemistry (IHC), the hydrated sections were blocked with hydrogen peroxide for 20 minutes before pepsin treatment. Next, the sections were blocked with 5% BSA for 1 hour at room temperature and incubated the with caspase-3, COX-2, and MMP-3 (1:200) primary antibodies overnight at 4°C, and further incubated with horseradish peroxidase (HRP)-linked secondary antibodies for 1 hour at room temperature. Then, 3,30-diaminobenzidine was used as a chromogenic agent. Immunohistochemical evaluations of OA were performed by three individuals using the ImageJ software (NIH, USA) to conduct optical density analysis. All sections were photographed and observed using an Olympus microscopic imaging system (model BX43, Olympus, Tokyo, Japan).

### Collection, isolation and culture of rat articular chondrocytes

This study was approved by the Institutional Animal Care and Use Committee of Zhejiang University (Hangzhou, China). Articular cartilage was obtained from the knees and hips of six-week-old Sprague Dawley (SD) rats (Zhejiang Academy of Medical Sciences Hangzhou China). The cartilage was cut into 1-mm3 pieces. Chondrocytes were isolated by digestion with 0.2% collagenase II in DMEM at 37°C for 2 h. The isolated cells were cultured in tissue culture flasks in DMEM supplemented with 10% FBS, 100 U/mL penicillin, and 100 μg/mL streptomycin in a 5% CO2 atmosphere at 37°C. When 80% confluence was observed, the chondrocytes were passaged and the cells between passages 2 and 4 were used for the subsequent experiments.

### Nesfatin-1 experimental concentration determination

After reaching 80% confluence, the chondrocytes were treated with DMEM/F12 with 1% FBS containing IL-1β (10 ng/mL) for 2 h. Then, the cells were treated with nesfatin-1 at 0, 0.1, 1, and 10 ng/mL for additional 24 h before using the CCK-8 assay to assess cell viability. The optical absorbance of the resulting colored solution was quantified at 450 nm using a spectrophotometer (Thermo). The optimal concentration of 10 ng/mL for nesfatin-1 was chosen for the subsequent experiments.

### Cell apoptotic rate detection by flow cytometry

The apoptotic rate of cells was evaluated using an Annexin V-FITC apoptosis detection kit (eBioscience, San Diego, CA, USA) according to the manufacturer’s instructions. Following the treatment stated earlier, the cells were washed with ice-cold PBS and harvested using a trypsin-EDTA solution. After centrifugation, the supernatant was discarded, and the cells were incubated in buffer containing annexin V- FITC and PI for 5 min at room temperature in the dark. Subsequently, the apoptotic cells were analyzed.

### Nitric oxide and PGE2 measurements

The cell supernatant of the culture medium was collected for the measurement of NO and PGE2. In brief, Griess reaction was used to assess the nitrite levels, and an enzyme-linked immunosorbent assay (ELISA) was used to measure PGE2 production according to the manufacturer’s instructions (R&D Systems, Minneapolis, MN, USA). All assays were performed in triplicate.

### Total RNA isolation and quantitative real-time polymerase chain reaction (PCR)

Intracellular RNA was extracted from a primary culture of chondrocytes using Trizol reagent (Invitrogen, Carlsbad, CA, USA) according to the manufacture’s protocol. Furthermore, it was reverse-transcribed to DNA templates using Malone murine leukemia virus reverse transcribe DNA synthesis kit (Promega, Madison, WI, USA) according to the manufacturer’s instructions. The reaction was conducted at 37°C for 15 min, 85°C for 5 sec, and then was terminated at 4°C. Quantitative altimeter PCR was performed with an IQ SYBR Green supermix PCR kit with the bi-cycle apparatus system (Bio-Rad, Hercules, CA, USA) as follows: 30 sec at 95°C for the initial denaturation, then 40 cycles of 15 sec at 95°C, 32 sec at 60°C, and 1 min at 72°C followed by 5 min at 72°C. The relative eRNA expression was assessed using the 2ΔΔCT method. The primers used are listed in Table 1.

**Table 1:** Primer sequences used in this study.

Gene*	**Genbank Accession**	**Forward**	**Reverse**	**Size (bp)**	**Tm**	**Self-complementarity**
Rat MMP3	NM_133523.3	CAGGCATTGGCACAAAGGTG	GTGGGTCACTTTCCCTGCAT	110	60°C	F:4
						R:4
Rat MMP9	NM_031055	GCAAACCCTGCGTATTTCCAT	GATAACCATCCGAGCGACCTTT	81	60°C	F:3
						R:3
Rat MMP13	NM_133530	CAACCCTGTTTACCTACCCACTTAT	CTATGTCTGCCTTAGCTCCTGTC	85	60°C	F:3
						R:5
Rat COX2	NM_017232.3	ATTCTTTGCCCAGCACTTCACT	CCTCTCCACCGATGACCTGATA	185	60°C	F:3
						R:4
Rat IL-6	NM_012589.2	AGCGATGATGCACTGTCAGA	GGAACTCCAGAAGACCAGAGC	127	60°C	F:5
						R:4
Rat GAPDH	NM_017008.4	CACCCAGCCCAGCAAGGATA	TCCTGTTGTTATGGGGTCTGG	129	60°C	F:2
						R:2
Rat Col2a1	NM_012929.1	GCCAGGATGCCCGAAAATTAG	GTCACCTCTGGGTCCTTGTTC	128	60°C	F:4
						R:3
Rat ADAMTS5	NM_198761.1	AGTACAGTTTGCCTACCGCC	CGTTAGGTGGGCAGGGTATG	116	60°C	F:4
						R:2

Rat means species is rat.

### Western blot analysis

In order to determine the effect of nesfatin-1 on IL-1β-induced apoptosis, primary chondrocytes were collected and lysed in lysis buffer, while cell debris were removed by centrifugation. The protein concentrations were measured using a bicinchoninic acid (BCA) protein assay kit (Pierce Biotechnology, Rockford, IL, USA). An aliquot of 20 μg of total proteins from each sample was subjected and separated by 10% sodium dodecyl sulfate-polyacrylamide gel electrophoresis (SDS-PAGE). Then, the proteins were blotted onto polyvinylidene fluoride (PVDF) membranes and blocked for 1 h with 2.5% skimmed milk at ambient temperature. The membranes were incubated with primary antibodies against Bcl-2 (dilution 1:500), GAPDH (dilution 1:10000), Bax, procaspase-3, cleaved caspase-3, PARP, cleaved-PARP, MMP-3, MMP-9, MMP-13, iNOS, COX-2, Col2a1, ADAMTS5, pp65, p65, pp38, p38, pJNK, and JNK overnight at 4°C. All the above primary antibodies were used at a 1:1,000 dilution, except for ADAMTS5, which was used at a dilution of 1:250. The membranes were subsequently washed thrice with Tris-buffered saline with Tween (TBST) and were incubated with horseradish peroxidase (HRP)-conjugated goat anti-rabbit and anti-mouse secondary antibodies (1:1,000; cat. nos., A0208 and A0216; Beyotime Institute of Biotechnology) for 2 h. Then, they were again washed thrice with TBST. The density (specific binding) of each band was measured using the SuperSignal® West Dura Extended Duration Substrate (Pierce, USA) with X-ray film exposure (Kodak, Hangzhou, China).

### Animal experiments

Forty-five SD rats (200–250 g; males; 6 weeks old) were obtained from the Animal Center of the Zhejiang Academy of Medical Sciences. With the approval of the Ethics Committee of the Second Affiliated Hospital, Zhejiang University School of Medicine, Hangzhou, China, an experimental OA model was prepared. For medial meniscectomy (MM) in the bilateral knee joints, the rats were randomly divided into three groups. Fifteen rats were used as controls, which received a sham-operation. In the treatment group (nesfatin-1 group), 50 ul solution (10 ng/ml) was injected intraarticularly in OA rats every 7 days starting at 1-week post-surgery, whereas the rats in the OA group received an equal volume of vehicle. After 6 weeks of treatment, all rats were sacrificed, and the knees were preserved in 4% paraformaldehyde solution [46].

### Histological examination

The samples of each group were fixed in 4% paraformaldehyde, decalcified with 10% formic acid for three months, buffered at pH 7.4, dehydrated through a series of ethanol solutions, embedded in paraffin, cut into 3-*μ*m-thick sections, and stained with safranin O-fast green. The Mankin score was used to define the degree of histological changes in the samples [47]. Three independent researchers assessed the extent of histological cartilage damage in a blinded manner. Other specimens used for IHC staining followed the same protocol as that for human cartilage. The primary antibody of Col2a1 was used at a dilution of 1:200 and ADAMTS5 was used at a dilution of 1:50.

### Statistical analysis

All the experiments were repeated three times. The SPSS 22.0 software was used for data analysis. Data were expressed as mean values ± SD. One-way analysis of variance followed by Tukey's post hoc test was used to compare differences between groups. The p values < 0.05 were considered statistically significant.
